# Unilateral Acute Renal Ischemia-Reperfusion Injury Induces Cardiac Dysfunction through Intracellular Calcium Mishandling

**DOI:** 10.3390/ijms23042266

**Published:** 2022-02-18

**Authors:** Carolina Victoria Cruz Junho, Laura González-Lafuente, José Alberto Navarro-García, Elena Rodríguez-Sánchez, Marcela Sorelli Carneiro-Ramos, Gema Ruiz-Hurtado

**Affiliations:** 1 Center of Natural and Human Sciences (CCNH), Federal University of ABC, Santo André 09210-580, SP, Brazil; carolina.junho@gmail.com; 2 Cardiorenal Translational Laboratory, Institute of Research Imas12, Hospital Universitario 12 de Octubre, Community of Madrid, 28041 Madrid, Spain; laura.gonzlafuente@gmail.com (L.G.-L.); jalbertong@gmail.com (J.A.N.-G.); elena.rodsanchez@gmail.com (E.R.-S.); 3 CIBER-CV, Hospital Universitario 12 de Octubre, 28041 Madrid, Spain

**Keywords:** renal ischemia and reperfusion, intracellular calcium handling, adult cardiomyocyte, cardiorenal syndrome, acute renal failure

## Abstract

Background: Acute renal failure (ARF) following renal ischemia-reperfusion (I/R) injury is considered a relevant risk factor for cardiac damage, but the underlying mechanisms, particularly those triggered at cardiomyocyte level, are unknown. Methods: We examined intracellular Ca^2+^ dynamics in adult ventricular cardiomyocytes isolated from C57BL/6 mice 7 or 15 days following unilateral renal I/R. Results: After 7 days of I/R, the cell contraction was significantly lower in cardiomyocytes compared to sham-treated mice. It was accompanied by a significant decrease in both systolic Ca^2+^ transients and sarco/endoplasmic reticulum Ca^2+^-ATPase (SERCA_2a_) activity measured as Ca^2+^ transients decay. Moreover, the incidence of pro-arrhythmic events, measured as the number of Ca^2+^ sparks, waves or automatic Ca^2+^ transients, was greater in cardiomyocytes from mice 7 days after I/R than from sham-treated mice. Ca^2+^ mishandling related to systolic Ca^2+^ transients and contraction were recovered to sham values 15 days after I/R, but Ca^2+^ sparks frequency and arrhythmic events remained elevated. Conclusions: Renal I/R injury causes a cardiomyocyte Ca^2+^ cycle dysfunction at medium (contraction-relaxation dysfunction) and long term (Ca^2+^ leak), after 7 and 15 days of renal reperfusion, respectively.

## 1. Introduction

Acute renal failure (ARF) caused by renal ischemia-reperfusion (I/R) injury is a complex clinical entity related to a variety of clinical and surgical settings, including sepsis and kidney transplantation [1,2,3], and is characterized by the suspension of blood flow to the kidney followed by restoration of perfusion and re-oxygenation. While ARF is correlated with high morbidity and mortality [4], and with an incidence ranging from 5% to up to 30% in patients treated in intensive care units [5], the renal failure per se is not commonly a primary cause of death, and it has been proposed that injury to extrarenal organs might explain the dismal outcome for patients [6]. Indeed, several studies have identified cardiac involvement as contributing to disease-related morbidity/mortality following I/R injury [7].

The complex physiological relationship that exists between the kidneys and the heart has long been appreciated, and damage to one organ may induce damage to the other, which has been termed the cardiorenal syndrome. This coexistence of cardiac and renal dysfunction has been classified into 5 types of cardiorenal syndrome based on the initial injured organ and the range of the conditions (acute or chronic) [8]. According to this classification scheme, acute cardiorenal syndrome type 3 is defined as cardiac injury secondary to ARF and is commonly observed after transplantation and use of nephrotoxic or cardiotoxic drugs [8].

Renal ischemia typically occurs in the setting of renal infarction, sepsis or kidney transplantation. It is often followed by an inflammatory state, redox imbalance and changes to the structure and function of renal cells, including apoptosis [9]. These features are also evident in cardiac tissue [10,11,12,13]. Previous studies from the group have already evidenced the importance of the first two weeks of renal reperfusion in the development of cardiac outcomes. After 7 days of renal I/R, we find a peak of systemic inflammation (maximum increase in inflammatory cytokines, TNF-α, IFN-γ and IL-1β), electrophysiological changes in the heart (increased QT and QTc intervals) [10] and an increase in NOX and NOS activities, along with increased cardiac cell damage [13]. After 15 days of renal I/R, a concentric-type cardiac hypertrophy and electrical alterations were observed, given the increase of hypertrophy biomarkers such as B-type natriuretic peptide (BNP) and α-actin [10,14] together with the increase of the LV mass [10] and prolongation of the QJ interval [15]. Moreover, ARF progression to acute kidney disease triggers cardiac injury, including heart failure (HF) development, because of the volume overload, inflammatory activation and metabolic disturbances in uremia [16].

Heart function is tightly controlled by the intracellular Ca^2+^ handling that mediates membrane excitation and contraction (EC)-coupling during the cardiomyocyte contraction-relaxation. EC-coupling transforms electrical signals into mechanical force, which relies on tightly coordinated Ca^2+^ release and reuptake in cardiomyocytes [17]. Cardiac muscle contraction is initiated by the depolarization of the membrane of cardiomyocytes, activating transient Ca^2+^ entry through L-type Ca^2+^ channels from the extracellular medium. This small increase in cytosolic Ca^2+^ concentration triggers a pronounced release of Ca^2+^ from the sarcoplasmic reticulum (SR), given the opening of ryanodine receptors type 2 channels (RyR_2_), in a physiological process termed Ca^2+^-induced Ca^2+^ release [15], inducing cardiomyocyte contraction. Relaxation occurs when cytosolic Ca^2+^ is transported back from the sarcoplasm to the SR. This process occurs principally by Ca^2+^-ATPase 2a pump (SERCA_2a_ Ca^2+^) re-uptake and also by Ca^2+^ extrusion to the extracellular medium by the Na^+^/Ca^2+^ exchanger [18]. Alterations in EC-coupling and Ca^2+^ handling are related to direct cardiac injury during HF by stimulating ventricular dysfunction and arrhythmia [18]. While defects in intracellular Ca^2+^ homeostasis are well known during HF, playing a principal role in causing mechanical dysfunction and arrhythmia, little is known about the potential cardiac Ca^2+^ imbalance in ARF. 

Deciphering the intracardiomyocyte alterations linked to Ca^2+^ handling would be clinically useful to better understand the extrarenal physiological consequences of ARF on the heart and may allow early intervention focused on protecting the myocardium in patients after I/R injury. Accordingly, the aim of the present study was to investigate whether ARF triggers relevant cardiac features related to cardiomyocyte dysfunction and intracellular Ca^2+^ handling in an experimental model of unilateral I/R injury for 7 and 15 days.

## 2. Results

### 2.1. Renal Ischemia and Reperfusion Induces Contractile Dysfunction

We employed an experimental model of ARF to comprehensively analyze the cardiac consequences of acute unilateral renal I/R through the occlusion of the left renal pedicle. Macroscopic parameters of each experimental group are shown in Table 1. Left kidney weight/ body weight (LKW/BW) and left kidney weight/ tibia length (LKW/TL) ratios were significantly lower in 15 d I/R mice than in the other experimental groups (*p* < 0.001). In contrast to LKW, the right kidney weight (RKW)/BW ratio was significantly higher in the 7 d I/R and 15 d I/R groups than in the sham group (*p* < 0.05 and *p* < 0.01, respectively), and RKW/TL was significantly increased in the 15 d I/R group (*p* < 0.05). These results indicate hypertrophy of the right kidney, likely because of compensational mechanisms to maintain renal function. In the same line, the HW/BW and HW/TL ratios were significantly higher in mice after 7 (*p* < 0.05) and 15 (*p* < 0.05 and *p* < 0.01) days of renal I/R than in sham mice. We used both BW and TL in order to ensure that we normalized with respect to the growth of the animal (TL) and not only with BW. As the tibia is a non-variable measurement at short-term, the changes are more accurately quantified by relating heart/kidney weight to tibia length than to body weight after the I/R surgery.

We next examined renal damage by analyzing the plasma levels of urea, BUN, FGF-23 and phosphates (Table 2). Urea and BUN were significantly higher in the 7 d I/R group than in the sham group (*p* < 0.05, respectively). FGF-23 levels were significantly higher in the 15 d I/R group than in the sham group (*p* < 0.01), and FGF-23 levels were also significantly elevated when compared with levels in the 7 d I/R group (*p* < 0.01). Likewise, phosphate levels were significantly higher in the 15 d I/R group than in the 7 d I/R group (*p* < 0.05).

**Figure 1:**
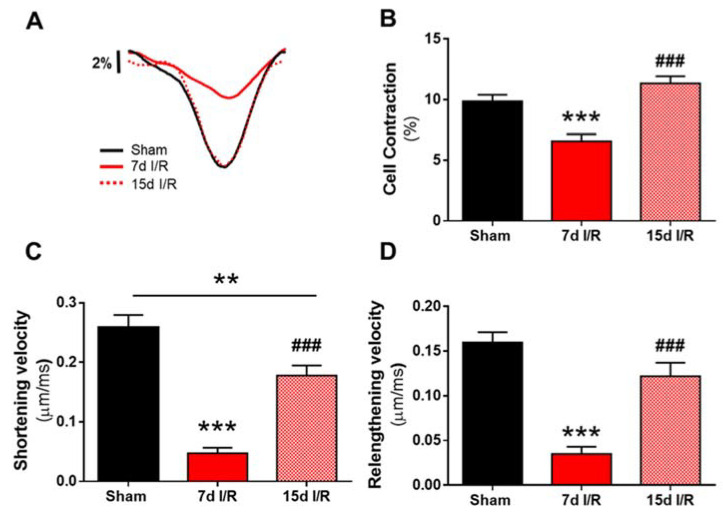
Contractility of electrically-stimulated cardiomyocytes after renal ischemia and reperfusion. (**A**) Cell contraction profiles of cardiomyocytes from sham-treated mice (black line) and from mice subjected to ischemia and reperfusion (I/R) for 7 (red line) and 15 (red discontinuous line) days. (**B–D**) Cell contraction (**B**), shortening (**C**) and re-lengthening (**D**) velocities. Cardiomyocytes were electrically stimulated at 2 Hz. Data are expressed as mean ± SEM. ** *p* < 0.01, *** *p* < 0.001 versus sham, ^###^ *p* < 0.001 versus 7 d I/R; sham (*n* = 73 cells, *N* = 10 mice), 7 d I/R (*n* = 39 cells, *N* = 10 mice) and 15 d I/R (*n* = 90 cells, *N* = 6 mice).

Subsequently, we investigated whether renal I/R could trigger changes to cardiomyocyte contractility using Fluo 3-AM loaded isolated adult ventricular cardiomyocytes. Representative cell-shortening profiles of cardiomyocytes from the three experimental groups are shown in Figure 1A. Cardiomyocyte contraction was significantly lower in the 7 d I/R group than in the sham group (*p* < 0.001; Figure 1B), and this was accompanied by significantly slower shortening and lengthening velocities (*p* < 0.001; Figure 1C,D). Notably, cell contraction and both shortening and lengthening velocities were recovered 15 days after renal I/R (Figure 1A), and all parameters were both significantly higher than those observed in cells from 7 d I/R mice (*p* < 0.001) and were closer to those observed in cardiomyocytes from sham mice (Figure 1B–D). Similar results have been previously observed in cardiac function in this experimental model of cardiorenal damage, in which 15 d I/R did not change echocardiographic parameters as shortening fraction, ejection fraction or cardiac output [10].

### 2.2. Systolic Ca^2+^ Release Deteriorates 7 Days following Renal Ischemia and Reperfusion 

**Figure 2:**
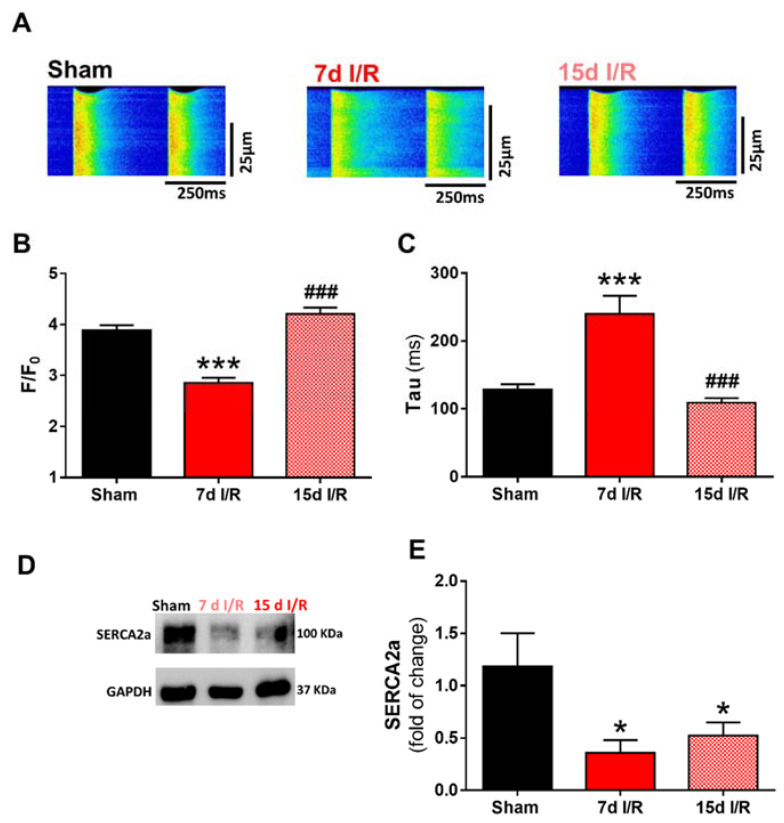
Systolic Ca^2+^ transient release in electrically stimulated cardiomyocytes. (**A**) Representative line-scan images of cardiomyocytes from sham-treated mice and from mice subjected to ischemia and reperfusion (I/R) for 7 and 15 days. Cardiomyocytes were electrically stimulated at 2 Hz. (**B**) Mean peak fluorescence Ca^2+^ transients (F/F_0_). (**C**) Decay time constant, Tau (ms). Data are expressed as mean ± SEM. *** *p* < 0.001 versus sham, ^###^ *p* < 0.001 versus 7 d I/R; sham (*n* = 80 cells, *N* = 10 mice), 7 d I/R (*n* = 61 cells, *N* = 10 mice), and 15 d I/R (*n* = 91 cells, *N* = 6 mice). (**D**) Representative immunoblots of SERCA_2a_ and GAPDH and (**E**) quantification of all experiments corresponding to cardiac protein expression. Data are presented as fold of change of sham levels ± SEM (sham = 9; 7 d I/R = 8; 15 d I/R = 8). * *p* < 0.05 versus sham.

Given the strong link between cardiac contractile dysfunction and alterations in intracellular Ca^2+^ handling and the release of Ca^2+^ from the SR, we next analyzed systolic Ca^2+^ release by measuring Ca^2+^ transients. Representative line-scan Ca^2+^ images obtained from Fluo-3AM-loaded cardiomyocytes during electrical stimulation at 2 Hz are shown in Figure 2A, and correspond to the sham (left panel), 7 d (medium panel) or 15 d (right panel) renal I/R groups. Analysis revealed that the amplitude of the intracellular Ca^2+^ transients (measured as the peak F/F_0_) was significantly lower in cardiomyocytes from the 7 d I/R mice than from the equivalent sham mice (*p* < 0.001; Figure 2B), and the decline recovered significantly after 15 days of renal injury (*p* < 0.001; Figure 2B). The time constant of Ca^2+^ transients decay (Tau) in cardiomyocytes was significantly longer in the 7 d I/R group than in the sham group (*p* < 0.001; Figure 2C), indicating a slower decay, but recovered 15 days after renal I/R (*p* < 0.001; Figure 2C). These effects on Ca^2+^ kinetics indicate a decline in the systolic Ca^2+^ peak, with prolongation of the duration of systolic Ca^2+^ transients and compromised re-pumping of Ca^2+^ back into the SR in mice after 7 d of renal I/R. SERCA_2a_ expression were significantly decreased after renal I/R (Figure 2D,E).

**Figure 3:**
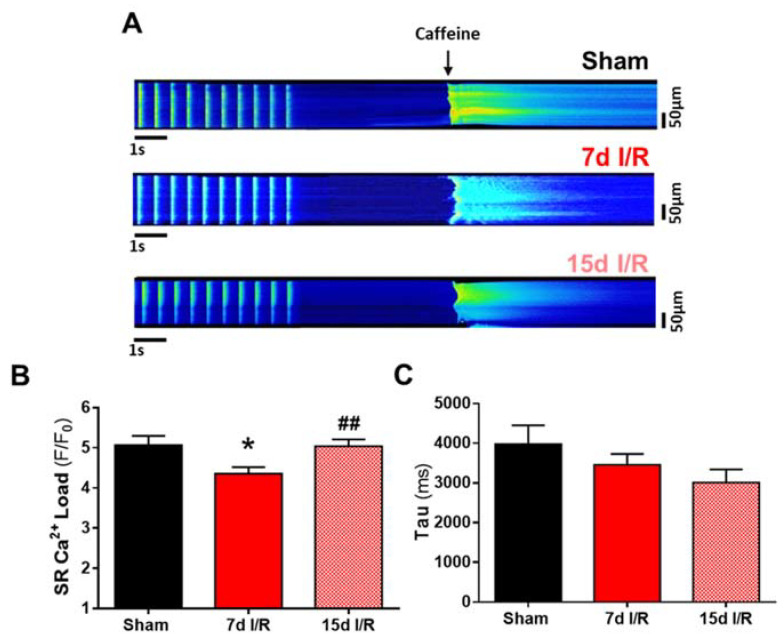
Sarcoplasmic reticulum Ca^2+^ load analysis on electrically stimulated cardiomyocytes. (**A**) Representative line-scan images of caffeine-evoked Ca^2+^ transients in cardiomyocytes from sham-treated mice and from mice subjected to ischemia and reperfusion (I/R) for 7 and 15 days. Cardiomyocytes were electrically stimulated at 2 Hz. (**B**) Mean values of the transient amplitude evoked by caffeine (peak fluorescence calcium, F/F_0_) in stimulated cardiomyocytes. (**C**) Decay time constant, Tau, in milliseconds. Data are expressed as mean ± SEM. * *p* < 0.05 versus sham, ^##^ *p* < 0.01 versus 7 d I/R; sham (*n* = 37 cells, *N* = 10 mice), 7 d I/R (*n* = 59 cells, *N* = 10 mice), and 15 d I/R (*n* = 73 cells, *N* = 6 mice).

We next questioned whether the changes in systolic Ca^2+^ release are related to alterations in SR Ca^2+^ load. Applying caffeine to isolated Fluo-3-loaded cardiomyocytes, Ca^2+^ stores from the SR were depleted. Representative line-scan images of caffeine-evoked Ca^2+^ transients are shown in Figure 3 and correspond to the sham (upper panel), 7 d (medium panel) or 15 d (bottom panel) I/R groups. Results showed that the amplitude of caffeine-evoked Ca^2+^ transients in cardiomyocytes was significantly lower in the 7 d I/R group than in the sham group (*p* < 0.05; Figure 3B), and that it was re-established in the 15 d I/R group (*p* < 0.05; Figure 3B). This occurred without significant changes in the rate of the decay time constant, Tau, in caffeine-exposed cardiomyocytes in any experimental group (Figure 3C).

### 2.3. Acute Renal Failure Induces Diastolic Ca^2+^ Release from the Sarcoplasmic Reticulum and Triggers Pro-Arrhythmic Ca^2+^ Events 7 Days of Renal Ischemia and Reperfusion That Remain Elevated after 15 Days

**Figure 4:**
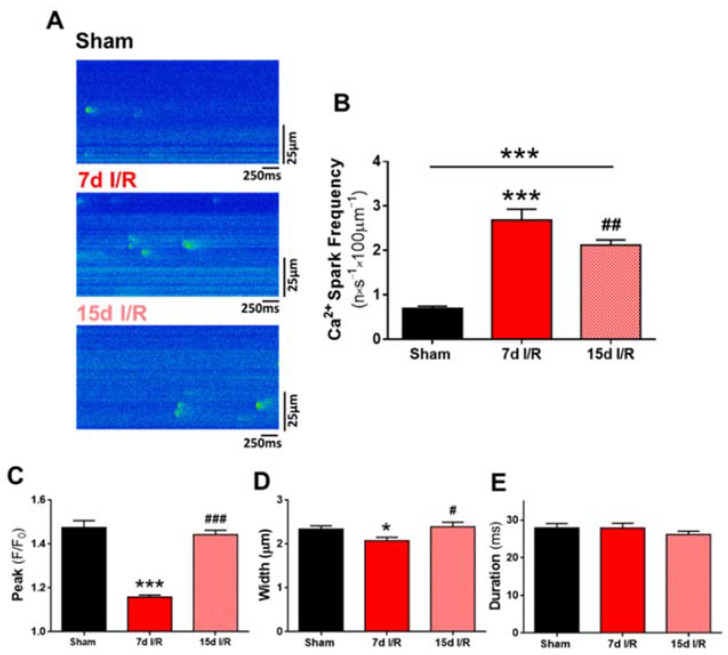
Diastolic Ca^2+^ leak in cardiomyocytes after renal I/R. (**A**) Representative line-scan images of Ca^2+^ sparks in quiescent cardiomyocytes from sham-treated mice and from mice subjected to ischemia and reperfusion (IR) for 7 and 15 days. (**B**) Frequency of Ca^2+^ sparks (presented in *n*·s^−1^·100 µm^−1^). (**C**) Mean peak of fluorescence Ca^2+^ sparks (F/F_0_). (**D**) Full width at half maximum of fluorescence of sparks (µm). (**E**) Full duration at half maximum of fluorescence Ca^2+^ sparks (ms). Data are expressed as mean ± SEM. * *p* < 0.05, *** *p* < 0.001 versus sham; ^#^ *p* < 0.05, ^##^ *p* < 0.01 versus and ^###^ *p* < 0.001 versus 7d I/R. sham (*n* = 57 cells, N = 10 mice), 7 d I/R (*n* = 43 cells, *N* = 10 mice) and 15 d I/R (*n* = 85 cells, *N* = 6 mice).

As alterations in SR Ca^2+^ load are frequently associated with changes in diastolic Ca^2+^ release, we next analyzed the frequency and the kinetic property of spontaneous Ca^2+^ sparks. Representative line-scan images of no stimulated cardiomyocytes are shown in Figure 4A and correspond to the sham (upper panel), 7 d (medium panel) and 15 d (bottom panel) I/R groups. Results showed that the frequency of Ca^2+^ sparks was significantly higher in cardiomyocytes at 7 and 15 days after ARF (Figure 4B; *p* < 0.001) than in cells from sham mice. Analysis of the morphological properties of Ca^2+^ sparks showed that the amplitude (peak F/F_0_) and full width was significantly lower in cardiomyocytes from 7 d I/R mice than from sham mice (Figure 4C,D; *p* < 0.001, *p* < 0.05, respectively). Both parameters recovered 15 days after renal I/R and were similar to those of sham cardiomyocytes (Figure 4C,D; *p* < 0.001, *p* < 0.05, respectively vs. 7 d I/R). No differences were found in the duration of Ca^2+^ sparks between the three experimental groups (Figure 4E).

**Figure 5:**
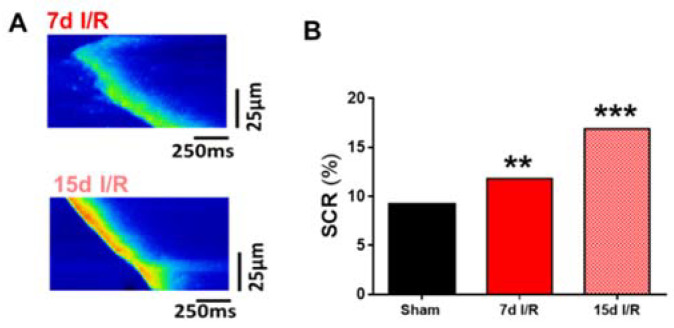
Spontaneous Ca^2+^ release in cardiomyocytes after renal I/R. (**A**) Representative line-scan images of cardiomyocytes from mice subjected to ischemia and reperfusion (I/R) for 7 and 15 days. (**B**) Spontaneous Ca^2+^ release measured as pro-arrhythmogenic events in cardiomyocytes. Data are presented as % of total analyzed cells. Data are expressed as mean of continuous variables after Fisher test. ** *p* < 0.01, *** *p* < 0.001 versus sham; sham (*n* = 56 cells, *N* = 4 mice), 7d I/R (*n* = 50 cells, *N* = 3 mice), 15d I/R (*n* = 99 cells, *N* = 6 mice). In this protocol each cell was recorded 8 consecutive times for 1.5 s. Statistical analyses were performed per scanned-file.

We next analyzed spontaneous Ca^2+^ waves and automatic Ca^2+^ transients during diastole, which were quantified as spontaneous Ca^2+^ release (SCR) in quiescent cardiomyocytes. Two representative examples of Ca^2+^ waves are shown in Figure 5A and correspond to cardiomyocytes isolated 7 (upper panel) and 15 (bottom panel) days after I/R. Quantification of the spontaneous Ca^2+^ events revealed that the occurrence of SCR was significantly higher after ARF, both at 7 (Figure 5B; *p* < 0.01) and 15 (Figure 5B; *p* < 0.001) days. There is a direct association between altered intracellular Ca^2+^ handling and a predisposition to fatal ventricular arrhythmias.

**Figure 6:**
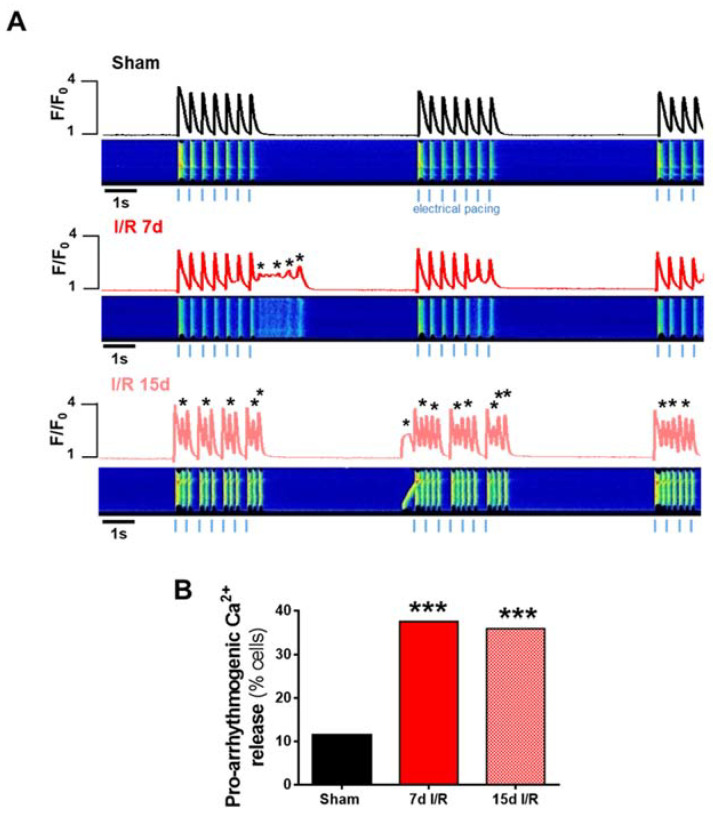
Pro-arrhythmogenic events in electrically stimulated cardiomyocytes. (**A**) Representative line-scan images and contraction profiles of cardiomyocytes from sham-treated mice and from mice subjected to ischemia and reperfusion (IR) for 7 and 15 days. Cardiomyocytes were electrically stimulated at 2 Hz in 7-pulse sequences. * Represents arrhythmic Ca^2+^ events (spontaneous abnormal release of Ca^2+^ as waves or automatic contractions). (**B**) Proarrhythmogenic Ca^2+^ release following the arrhythmia protocol in cardiomyocytes. Data presented as % of total analyzed cells with events. Data are expressed as mean of continuous variables after Fisher test. *** *p* < 0.001 versus sham; sham (*n* = 113 cells, *N* = 5 mice), 7d I/R (*n* = 77 cells, *N* = 7 mice), 15d I/R (*n* = 150 cells, *N* = 6 mice). In this protocol, each cell was recorded once for 30 s. Statistical analyses were performed per cell.

Accordingly, we next evaluated the presence of pro-arrhythmic behavior as spontaneous Ca^2+^ waves or transients evoking extra-contraction and missing Ca^2+^ transients in ventricular cardiomyocytes stimulated at 2 Hz for three cycles, each separated with a non-pacing period. Representative line-scan images of a normal cellular response corresponding to a cardiomyocyte from a sham-treated mouse is shown in Figure 6A (upper panel), and cardiomyocytes from mice after 7 and 15 days are shown in the middle and lower panels, respectively. Results showed that automatic Ca^2+^ dependent pro-arrhythmic events were significantly greater in cardiomyocytes from the renal I/R group (more than 30% of cells showed events) than cardiomyocytes from sham mice, both at 7 and 15 days following renal I/R (Figure 6B; *p* < 0.001).

## 3. Discussion

In the present study, we show that ARF induces intracellular Ca^2+^ mishandling, manifested as cardiomyocyte contractile dysfunction 7 days after left unilateral renal occlusion, which recovers 15 days after the renal I/R. Likewise, ARF increases the incidence of cardiomyocyte pro-arrhythmic Ca^2+^ events in the heart after 7 and 15 days in vitro, indicating that aberrant diastolic Ca^2+^ leak, and thus the risk of arrhythmic events, continues in the long term. The findings of this study are corroborated by other studies from our group that demonstrated the efficiency of renal I/R to promote arrhythmogenic events in vivo using the isoproterenol challenge after 15 days of reperfusion [15].

ARF is associated with an unacceptably high rate of mortality despite being recognized for more than 40 years [4]. ARF is considered a risk factor for the generation of cardiac disease, while HF is one of the most typical causes of death in patients with ARF [7,16]. As yet, the pathophysiological mechanisms of cardiac failure in ARF remain unresolved and identifying these mechanisms will be an important first step in reducing the high cardiovascular morbidity and mortality related to ARF. In this sense, the intracellular mechanisms governing Ca^2+^ handling are an important platform to investigate cardiomyocyte contractility. Even though they are very often described in other cardiac diseases, such as HF, the mechanisms surrounding Ca^2+^ handling dysfunction have not yet been studied much in the context of acute cardiorenal syndrome type 3. 

The present study shows—for the first time, to the best of our knowledge—relevant dysfunctional Ca^2+^ handling in ventricular cardiomyocytes in an experimental model of ARF at early (7 days) and later (15 days) stages following renal I/R injury. Our results establish an early impairment in cardiomyocyte contraction-relaxation processes after 7 days of renal I/R that is chiefly due to: (1) a decrease in systolic Ca^2+^ release, observed as a reduction in Ca^2+^ transient amplitude; (2) an increase in the decay time constant of Ca^2+^ transients; and (3) a depressed SR Ca^2+^ load. The Ca^2+^ handling abnormalities, together with the compromised contraction-relaxation cycle in cardiomyocytes observed 7 days post-renal I/R, are similar to what is observed in several models of HF [19,20,21]. Our data indicate that Ca^2+^ reuptake into the SR is impaired in ventricular cardiomyocytes after 7 days of renal I/R, which is considered an early phase of kidney injury. This could be explained by the reduction in expression or activity of SERCA_2a_. Similar to that which occurs in HF [21], the decline in SERCA_2a_ function might explain the depletion of Ca^2+^ stores in the SR, the lower transient amplitudes and the impaired cellular contractility. These activities recovered 15 days after renal I/R in mice and were similar to cardiomyocytes from sham mice. A plausible explanation for this recovery is that ARF after 15 days is considered a transition period between the acute and chronic state of post-renal injury, according to the guidelines of the Kidney Disease: Improving Global Outcomes (KDIGO) organization [22], and it is possible that the initial cardiac damage at this time, at least in terms of contractile-relaxation, is compensated for. Indeed, the heart outcomes concerning the Ca^2+^ mishandling in systole seem to be set before the 7th day. Analyzing the adult cardiomyocytes in previous timepoints could provide us a superior understand about the acuteness of the cardiac dysfunction after renal I/R.

An increase in SR Ca^2+^ leak during diastole is a known and shared Ca^2+^-dependent pathological mechanism associated with the HF progression and arrhythmias [17,23]. The Ca^2+^ leak is a phenomenon defined as an inappropriate release of Ca^2+^ from the SR [24]. In pathological conditions with direct damage of the heart as in HF or indirect as in ARF observed in the present study, there is a high release of Ca^2+^ from SR during diastole (manifested as a higher frequency of Ca^2+^ sparks, waves and spontaneous Ca^2+^ transients). This increased release of Ca^2+^ leak reduces its availability for the subsequent contraction, thereby impairing contractility as it was observed after 7 days of renal I/R. However, despite the evident cardiac improvement in terms of systolic Ca^2+^ handling and contraction after 15 days of renal I/R, diastolic Ca^2+^ leak remains abnormally elevated.

The increase in diastolic Ca^2+^ leak is potentially a pathological substrate for the induction of arrhythmias, as the released Ca^2+^ from the SR can diffuse to neighboring RyR_2_ clusters to induce pro-arrhythmogenic Ca^2+^ events that, together with automatic contractions, ultimately trigger ventricular arrhythmias. Gambardella et al. reinforced that the RYR_2_ represents the central target of many pathways dysregulated in cardiac pathological conditions, including metabolic disorders, ROS production and inflammation [24]. All these alterations are accompanied by alterations in Ca^2+^ handling and subsequent impairment in cardiac contraction. The study of stabilization of RYR_2_ seems to be the main target for clinical practice, being studied in models of genetic mutation in human stem cells derived cardiomyocytes [25] and as a target in HF [26]. Evidently, analyzing the RYR_2_ protein expression would be enlightening to provide a better understanding for the cardiorenal syndrome type 3 and its consequences to the heart.

A possible explanation for these results is that renal injury was sustained or worsened after 15 days of I/R, which is supported by the finding that the highest levels of FGF-23 were observed 15 days after renal I/R. FGF-23 is one of the most important components of mineral and bone metabolism and its excess is linked to the progression of renal dysfunction [27]. FGF-23 is a bone-produced hormone involved mainly in the response to an increase in parathyroid hormones, vitamin D or phosphorus. In the kidney, it binds to the FGF receptor and to its membrane co-receptor Klotho, to stimulate phosphaturia. Systemic FGF-23 levels rapidly increase as renal function declines, and this occurs before an increase in other markers, such as parathyroid hormone, vitamin D or even phosphorus, likely as a compensatory mechanism to prevent hyperphosphatemia after ARF [28,29]. In recent years, FGF-23 has also been established as an independent modulator of the heart [30,31,32]. Functionally, it has been demonstrated that FGF-23 triggers pro-arrhythmogenic cellular events in the HL-1 murine atrial cardiomyocyte cell line [33]. This might go some way to explain the clinical data showing that higher systemic FGF-23 levels are associated with a higher incidence of atrial fibrillation [34]. Moreover, it has recently been demonstrated that FGF-23 can induce relevant changes in Ca^2+^ handling in adult ventricular cardiomyocytes [31,35]. Analogous to our findings in ventricular cardiomyocytes from mice with renal I/R, the exposure to FGF-23 induced spontaneous diastolic spontaneous Ca^2+^ leak from the SR in the form of Ca^2+^ SCR and triggered in vitro pro-arrhythmogenic activity as automatic systolic Ca^2+^ transients and extra-contractions. Therefore, our results suggest that the vulnerability to ventricular arrhythmias at early and longer term after I/R injury might be explained, at least in part, by the elevated and maintained circulating FGF-23 levels.

**Figure 7:**
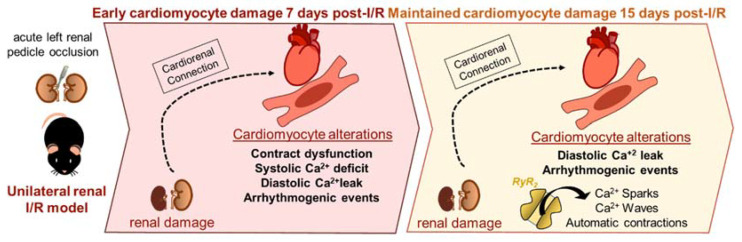
Schematic drawing of the take-home messages.

In conclusion, our study reveals a different pattern of cardiac damage at the medium and long terms after ARF, characterized by Ca^2+^ cycle dysfunction in ventricular cardiomyocytes. Despite the resolution of the initial contraction-relaxation dysfunction, the Ca^2+^ leak observed during diastole remains as an important sequela (Figure 7) that might have a significant impact on long-term cardiac outcomes such as fatal ventricular arrhythmias in patients with ARF. In fact, 7 and 15 days are important timepoints to observe the most important alterations in this ARF model, however, assess the Ca^2+^ behavior in other different timepoints was a limitation of the study. Evaluate cardiomyocytes of animals of previous and future reperfusion times would clarify the understand about the acuteness dysfunction observed after 7 days of renal I/R, as well as the consequence of maintained Ca^2+^ leak after 15 days, respectively.

Overall, our study provides a framework to better understand the mechanisms related to cardiac alterations and the elevated risk of major cardiovascular events including cardiac dysfunction and or fatal arrhythmias and cardiovascular mortality observed in patients with ARF. It may also serve as a basis for future pharmacological therapies focused on preventing the deleterious effects of mineral bone components in the uremic context and better managing fatal cardiac outcomes in patients with ARF.

## 4. Materials and Methods

### 4.1. Animal Procedures

The animal study was conducted following the recommendations of the Spanish Animal Care and Use Committee according to the Guidelines for Ethical Care and Welfare (2013/175) of experimental animals and the directive of the European Union (2010/63/EU) on the protection of animals used for scientific purposes. The General Directorate of Agriculture and the Environment of the Environment Council of Madrid approved the study (PROEX 186.5/20). Male C57BL/6 mice (6–8 weeks of age, 20–25 g) were purchased from Charles River Laboratories International (Brussels, Belgium). Mice were group housed in cages (maximum of five) at the Experimental Animal Center, Hospital Universitario 12 de Octubre. Mice were maintained on a 12-h light/dark artificial cycle, at a constant ambient temperature of 23–25 °C, with ad libitum access to water and a standard diet.

### 4.2. Experimental Renal Ischemia/Reperfusion

Mice underwent sham or I/R surgery using a previously described procedure [10,12,15]. Isoflurane 1.5% was used to anesthetize the mice. To simulate renal ischemia, the left renal pedicle was exposed after an abdominal incision, and was occluded using a microvascular clamp for 60 min. After this time, the clamp was removed, and the animals were sutured using silk thread. Meloxicam (0.06 mL/kg; single dose; s.c.) was used as analgesia after surgery. Mice were randomly divided into three groups: sham and renal ischemia with 7 (7 d I/R) or 15 days (15 d I/R) of reperfusion. These timepoints were selected considering the alterations previously studied by the group that demonstrate relevant cardiac alterations at the 7th and 15th day of renal I/R [10,13,14,15]. After the first week of reperfusion we observed the increase of inflammatory cytokines, cardiac cell damage and functional cardiac alterations [10,13,15]. After the second week, we observed the cardiac hypertrophy and the prevalence of electrical alterations [10,15]. Consequently, animals were euthanized 7 or 15 days following surgery and blood samples, heart and kidneys samples were collected for processing. Samples were maintained at −80 °C until analysis.

### 4.3. Biochemical Assays

The Urea Assay Kit (BioAssay Systems, Hayword, CA, USA) was used to measure plasmatic urea and blood urea nitrogen (BUN) levels. The Phosphate Assay Kit (Abcam, Cambridge, UK) was used to measure plasmatic phosphate. Lastly, the FGF-23 Dosing Kit (Immutopics, San Clemente, CA, USA) was used to measure plasmatic FGF-23 levels by ELISA. All assays were used according to the manufacturers’ recommendations.

### 4.4. Isolation of Adult Ventricular Cardiomyocytes

Mice were anesthetized with sodium pentobarbital-heparin (100 mg/kg, i.p.) and the heart was rapidly removed. It was connected to a Langendorff perfusion system through the ascending aorta [19]. A Ca^2+^-free Tyrode’s solution was used to retrograde perfuse the hearts. After that, the hearts were digested using a type II collagenase solution with 1 mg/mL (Worthington, Lakewood, NY, USA), as previously described [36]. Then, hearts were removed from the Langendorff apparatus and ventricles were cut into small pieces to disperse the isolated ventricular cardiomyocytes. Cell suspensions were filtered through a 250-μm nylon mesh and then pelleted by centrifugation for 3 min at 20× *g* and suspended in Tyrode’s solution. Only rod-shaped, excitable under electrical pacing and Ca^2+^-tolerant cardiomyocytes with clear cross-striations were selected and used for intracellular Ca^2+^ imaging studies.

### 4.5. Intracellular Ca^2+^ Imaging

An intracellular fluorescent calcium dye Fluo-3AM (5 µmol/L; Invitrogen, Carlsbad, CA, USA) was used to load the isolated ventricular cardiomyocytes. They were field stimulated by two electrodes at 2 Hz. Intracellular Ca^2+^ was imaged by confocal microscopy (Meta Zeiss LSM510, Jena, Germany. Objective 40×, 1.2 NA) at the speed of 1.5 ms/line (1000 lines per image) for recording Ca^2+^ transients, Ca^2+^ sparks and Ca^2+^ wave images, and at 3 ms/line (10,000 lines per image) for caffeine and arrhythmia protocols. All Ca^2+^ images were corrected for background fluorescence. For Ca^2+^ transients, the fluorescence value (F) was normalized by baseline fluorescence (F_0_) to obtain F/F_0_. The decay time constant of the Ca^2+^ transients, Tau, was obtained by adjusting the decay trace of the fluorescence to a single exponential. The measurement of the cell shortening was made using the difference of cardiomyocyte length between electrical stimulation and resting period. It was displayed as the percentage cell shortening length. The Ca^2+^ load of the SR was estimated in intact ventricular myocytes by a quick challenge with 10 mmol/L caffeine to deplete Ca^2+^ stores. Caffeine induces Ca^2+^ release by reducing the threshold for luminal Ca^2+^ activation of the RyR, causing a total Ca^2+^ release from intracellular Ca^2+^ stores [37]. This methodology was previously used by the group to measure the SR Ca^2+^ load [28,38,39]. To quantify spontaneous Ca^2+^ sparks, Fluo-3AM-loaded cardiomyocytes were maintained without electrical stimulation and Ca^2+^ spark regions were determined as the specific sites where the fluorescence signal briefly increased by at least four times the standard deviation of the fluorescence. Abnormal spontaneous Ca^2+^ release, manifested as Ca^2+^ waves or automatic Ca^2+^ transients without electrical stimulation, was quantified by the percentage of occurrence, and globally referred to as spontaneous calcium release (SCR). Three cycles of a 2 Hz electrical stimulation field were applied to record pro-arrhythmogenic activity. This consisted of seven pulses with a resting period in each cycle. Arrhythmic Ca^2+^ events were considered to be any spontaneous abnormal release of Ca^2+^ as waves, missing transients or automatic contractions corresponding to spontaneous Ca^2+^ transients during the protocol. All Ca^2+^ images were analyzed using home-made routines with IDL (Research Systems, Boulder, CO, USA) and Image J (National Institutes of Health, Bethesda, MD, USA) softwares.

### 4.6. Immunobloting

Total protein was isolated from hearts by lysing the tissue with an extraction buffer (0.05 M Tris, 0.32 M sucrose, 0.5% CHAPS, 0.5 μM okadaic acid, and the protease inhibitors 0.1 M PMSF, 12 μM leupeptin, 0.2 μM aprotinin and 0.5 M benzamidine, pH 7). Protein content was quantified using a BCA kit (Thermo Fisher Scientific, Waltham, MA, USA). Next, 20 μg of the extracted proteins was loaded in a 12% SDS-page gel and was electrophoresed for 180 min at a constant voltage of 120 V. Next, the proteins resolved on the gel were transferred onto a PVDF membrane using a semi-dry transfer system (Trans-Blot SD, Bio-Rad, Hercules, CA, USA). The membranes were blocked with 5% skimmed milk in TBST buffer (TBS, 0.1% Tween 20) for 1 h and incubated overnight at 4 °C with primary antibodies. After washing, the membrane was incubated with a corresponding peroxidase-conjugated secondary antibody for 1 h at room temperature. Finally, the membrane was analyzed using an ECL system (Thermo Fisher Scientific, Waltham, MA, USA). Protein bands were visualized using ImageQuant LAS4000 and band densities were measured using ImageJ software (National Institutes of Health, Bethesda, MD, USA). Primary antibodies used were anti-SERCA_2a_ antibodies at 1:2.000 dilution (Abcam, Cambridge, UK) and anti-GAPDH antibodies at 1:200,000 (Thermo Fisher Scientific, Waltham, MA, USA). Secondary antibodies used were anti-mouse at 1:5000 dilution (Santa Cruz Biotechnology, Dallas, TX, USA).

### 4.7. Statistical Analysis

Data are here stated as mean ± standard error of the mean (SEM). Statistical significance was accomplished applying an un-paired Student’s test, χ^2^ test or analysis of variance (ANOVA), with a Newman-Keuls multiple comparison test used when appropriate. It was performed with OriginPro 9.0 (OriginLab, Northampton, MA, USA) or GraphPad Prism v6.0 (GraphPad Software Inc., San Diego, CA, USA). Differences with *p*-values < 0.05 were considered statistically significant.

## Figures and Tables

**Table 1:** Macroscopic parameters in the ARF model 7 and 15 days following renal ischemia and reperfusion.

Parameters	Sham	7 d I/R	15 d I/R
BW (g)	24.0 ± 0.5	22.2 ± 0.6	23.2 ± 0.7
TL (mm)	16.6 ± 0.13	16.2 ± 0.12	16.2 ± 0.21
HW (g)	0.160 ± 0.005	0.177 ± 0.009	0.179 ± 0.007
LKW (g)	0.151 ± 0.004	0.148 ± 0.004	0.115 ± 0.006 ***, ^###^
RKW (g)	0.146 ± 0.009	0.167 ± 0.005	0.178 ± 0.007 *
HW/BW (mg/g)	6.7 ± 0.21	7.97 ± 0.37 *	7.77 ± 0.32 **
HW/TL (mg/mm)	96.9 ± 3.09	109.1 ± 6.15 *	110.2 ± 3.34 *
LKW/BW (mg/g)	6.3 ± 0.10	6.7 ± 0.28	5.0 ± 0.25 ***, ^###^
LKW/TL (mg/mm)	90.9 ± 2.08	91.1 ± 2.83	70.7 ± 3.28 ***, ^###^
RKW/BW (mg/g)	6.01 ± 0.37	7.56 ± 0.36 *	7.70 ± 0.35 **
RKW/TL (mg/mm)	88.3 ± 5.63	102.7 ± 3.68	109.2 ± 3.85 *

Morphometric data on kidney and heart weight and size in animals (*N* = 6–11) 7 or 15 days following renal ischemia and reperfusion (7d I/R, 15d I/R). Data are expressed as mean ± SEM. * *p* < 0.05, ** *p* < 0.01 versus sham; *** *p* < 0.001 versus sham; ^###^ *p* < 0.001 7d I/R. BW, body weight; TL, tibia length; HW, heart weight; LKW, left kidney weight; RKW, right kidney weight; I/R, ischemia and reperfusion.

**Table 2:** Biochemical parameters in the ARF model 7 and 15 days following renal ischemia and reperfusion.

Parameters	Sham	7 d I/R	15 d I/R
Urea (mg/dL)	40.9 ± 2.5	61.2 ± 8.9 *	51.6 ± 3.2
BUN	19.1 ± 1.2	28.6 ± 4.2 *	24.1 ± 1.5
FGF-23 (pg/mL)	186.6 ± 50.5	298.0 ± 65.2	759.7 ± 151.1 **^,^^##^
Phosphate (mg/dL)	6.05 ± 1.01	4.43 ± 1.01	9.33 ± 1.74 ^#^

Plasma levels of kidney function (urea, BUN and phosphate) and (FGF-23) in animals (*N* = 6–11) 7 or 15 days following renal ischemia and reperfusion (7 d I/R, 15 d I/R). Data are expressed as mean ± SEM. * *p* < 0.05, ** *p* < 0.01 versus sham; # *p* < 0.05, ## *p* < 0.01 versus 7 d I/R. BUN, blood urea nitrogen; FGF-23, fibroblast growth factor-23; I/R, ischemia and reperfusion.

## Data Availability

Not applicable.

## Abbreviations

ARF	acute renal failure
BUN	blood urea nitrogen
BW	body weight
Ca^2+^	calcium
CRS	cardiorenal syndrome
EC	excitation-contraction
FGF-23	fibroblast growth factor-23
HF	heart failure
HW	heart weight
I/R	ischemia and reperfusion
IRI	ischemia reperfusion kidney injury
LKW	left kidney weight
RKW	right kidney weight
RyR_2_	ryanodine receptor
SERCA_2a_	sarcoplasmic/endoplasmic reticulum Ca^2+^-adenosine triphosphatase 2a
SCR	spontaneous calcium release
SR	sarcoplasmic reticulum
TL	tibia length
